# Screening of mitochondrial-related biomarkers connected with immune infiltration for acute respiratory distress syndrome through WGCNA and machine learning

**DOI:** 10.1097/MD.0000000000041497

**Published:** 2025-03-07

**Authors:** Wei Sun, Su Tu

**Affiliations:** a Department of Emergency, Jiangnan University Medical Center, JUMC, Wuxi, Jiangsu Province, China.

**Keywords:** acute respiratory distress syndrome, immune cell, machine learning, mitochondria, WGCNA

## Abstract

Septic acute respiratory distress syndrome (ARDS) is a complex and noteworthy type, but its molecular mechanism has not been fully elucidated. The aim is to explore specific biomarkers to diagnose sepsis-induced ARDS. Gene expression data of sepsis alone and sepsis-induced ARDS were downloaded from public databases, and the differential immune cells and differential expressed genes between the 2 groups were screened. Weighted gene co-expression network analysis was used to identify immune cells-related module genes, and then integrated with mitochondrial genes to obtain common genes. Next, least absolute shrinkage and selection operator, random forest, and support vector machine-recursive feature elimination were utilized to construct a nomogram model. Meanwhile, the biological function and targeted drugs of biomarkers were analyzed. The abundance of 3 immune cells (macrophage, neutrophils, and monocytes) was significantly different between the 2 groups. Weighted gene co-expression network analysis and machine learning identified 5 biomarkers were up-regulated in ARDS and had diagnostic significance. Next, the nomogram based on these genes had good confidence and clinical application value. Gene set enrichment analysis showed that phenylalanine metabolism pathway was increased in ARDS samples and had positive correlation with diagnostic genes. Drug prediction analysis exhibited that chlorzoxazone, ajmaline, and clindamycin could target multiple diagnostic genes. Overall, the diagnostic signature screened in this study can effectively predict the possibility of ARDS in sepsis patients, which can deepen the understanding of ARDS pathogenesis and targeted therapy development.

**Key Points**

- Infiltration of macrophage, neutrophils, and monocytes was different in sepsis and ARDS.
- Five mitochondrial-related biomarkers were screened via WGCNA and machine learning.
- Nomogram developed by 5 biomarkers showed good predictive performance in ARDS diagnosis.
- Phenylalanine metabolism pathway was markedly activated in ARDS than in sepsis group.
- RT-qPCR confirmed the up-regulation of 5 genes in ARDS compared to sepsis.

## 1. Introduction

Acute respiratory distress syndrome (ARDS) is a clinical syndrome of diffuse pulmonary inflammation and non-cardiogenic pulmonary edema, which often leads to acute respiratory failure.^[1]^ It has been reported that ARDS occurs in 1/4 of all critically ill patients requiring mechanical ventilation.^[2]^ Despite significant advances in the clinical recognition and treatment of ARDS, it remains a life-threatening disease, with a mortality rate consistently in the range of 30% to 40%.^[3]^ ARDS is a heterogeneous disease, which is mainly manifested in clinical features, etiology, imaging, and biomarkers.^[4]^ These heterogeneities have greatly prevented the development of therapeutic approaches for ARDS. Among the ARDS subtypes, septic ARDS is a complex and noteworthy type.^[5]^ Sepsis is the most risk factor for ARDS, accounting for about 40% of ARDS cases.^[6]^ Meanwhile, the prognosis of patients with sepsis complicated with ARDS is worse than those with ARDS alone, usually accompanied by a higher mortality.^[7]^ Therefore, it is critical to pay attention to the risk of ARDS in patients with sepsis in clinical practice. Currently, the molecular mechanisms of sepsis-induced ARDS have not been fully elucidated, and effective therapeutic agents for ARDS are still lacking.^[8]^ Altogether, there is an urgent need to develop specific targeted therapeutic strategies for these patients.

Immune cells and cytokines are involved in different stages of ARDS. Briefly, microorganisms and pathogens invade the lung tissue and releases large amounts of inflammatory factors, which activate the recruitment of multiple effector cells (such as neutrophils, macrophages, and lymphocytes) into the alveolar airspaces, leading to an uncontrolled inflammatory cascade.^[9]^ These cells and secretions cause damage to the alveolar epithelial and endothelial barriers, leading to accumulation of edema fluid, which ultimately caused reduced lung function and severe lung injury.^[10,11]^ In addition, metabolic reprogramming of immune cells can trigger pro- and anti-inflammatory response, which are mainly determined by the metabolic activity of specific enzymes and phenotype of cells.^[12]^ However, the current understanding of the immune profile of patients with sepsis-induced ARDS remains limited.

Mitochondria are the energy source of cells, involved in biosynthesis, energy production, and signal transduction.^[13]^ Besides, mitochondria play a role in the repair of alveolar epithelial and endothelial barriers.^[14]^ Increasing evidences suggest that mitochondrial dysfunction is closely related to the pathological features of lung injury, including inflammatory factor release and immune cell infiltration.^[15,16]^ For example, the copy number of mitochondrial DNA (mtDNA) in peripheral blood of patients with ARDS is associated with adverse clinical outcomes and can be considered as a predictor of acute lung injury.^[17]^ In addition, during the development of ARDS, mitochondrial dysfunction in damaged epithelial/endothelial cells and leukocytes, which in turn generates large amounts of reactive oxygen species and releases mtDNA, activating the intrinsic immune response.^[18]^ It follows that factors associated with mitochondrial pathology may be targets for lung injury therapy. Unfortunately, the research of mitochondria-related elements in ARDS is still at a preliminary stage, and the link among ARDS, immune infiltration and mitochondria is unclear. Hence, it is necessary to understand the interaction between immune cells and mitochondria in ARDS, aiming to provide better options for the diagnosis and treatment of ARDS.

**Figure 1.**
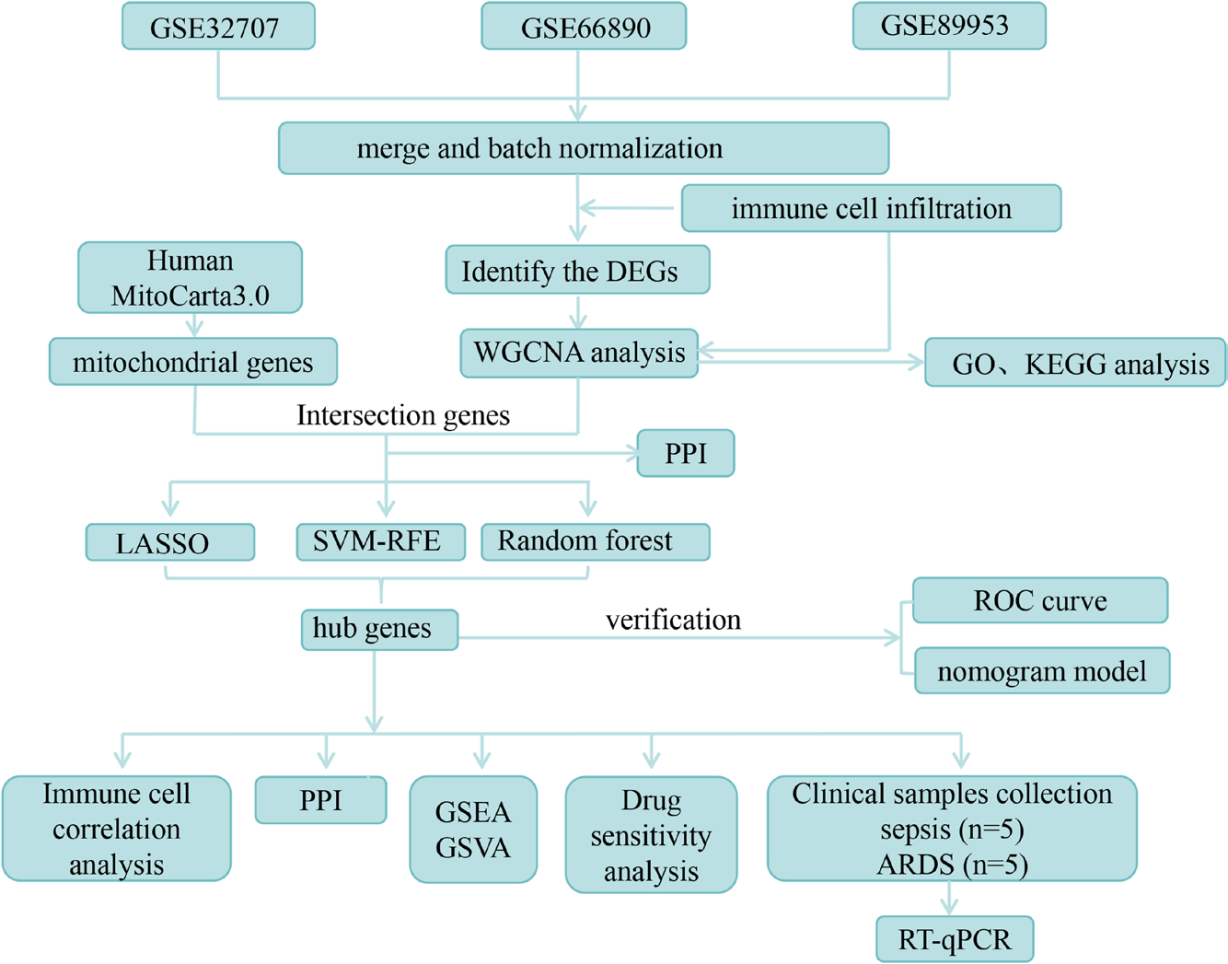
The workflow diagram of this study.

In this study, based on the expression profile pf ARDS and mitochondria-related genes from public databases, key genes related to sepsis-induced ARDS were screened through differential gene analysis, immune cell analysis, weighted gene co-expression network analysis (WGCNA), and machine learning algorithms. Moreover, the diagnostic model for ARDS was constructed. This work provides new ideas for the intervention and treatment of ARDS. The flowchart of this study was shown in Figure 1.

## 2. Materials and methods

### 2.1. Data collection and preprocessing

The ARDS-related databases were obtained from the Gene Expression Omnibus database. The datasets included in this analysis were required to meet the following criteria: (1) transcriptome expression profiles for ARDS were contained; (2) the sample size was >25; and (3) the test samples were from human whole blood. A total of 4 datasets met the inclusion criteria. Specially, GSE32707 included 31 ARDS and 58 sepsis samples; GSE66890 recorded 29 ARDS and 28 sepsis samples; GSE89953 contained 26 ARDS samples; as well as GSE10474 contained 13 ARDS and 21 sepsis. In this analysis, GSE32707, GSE66890, and GSE89953 served as the training set, while GSE10474 served as the validation set.

The mRNA probe expression matrix and platform annotation information corresponding to each dataset were downloaded, and the probes were converted into gene symbols. When multiple probes were applied to detect the same gene, the average of all probes were used as the expression value. Next, the R language sva package (version 3.36.0)^[19]^ was used to remove batch effects among 3 training sets, and then expression profiles were merged for subsequent analysis.

### 2.2. Evaluation of immune cell infiltration

CIBERSORT,^[20]^ a method for characterizing the cellular composition of complex tissues from gene expression profiles, was used to assess the proportion of immune cells in all samples. Then, the single-sample gene set enrichment analysis (ssGSEA) algorithm in R software was applied to determine the enrichment of 28 immune cells in the samples.^[21]^ Differences in the distribution of immune cells in ARDS and sepsis were compared using the Wilcoxon test.

### 2.3. Identification of differential expressed genes (DEGs)

The limma package (version 3.34.7) in R was used to analyze the differential expression of the ARDS and sepsis groups,^[22]^ and the corresponding *P* value and log FC values of genes were calculated. In addition, Benjamini–Hochberg method was employed to correct the multiple testing, and adjusted *P* value were obtained. The thresholds for DEGs were set as follows: adjusted *P* value < .05 and |log2FC| ≥ 1.

### 2.4. WGCNA

WGCNA is employed to discover gene sets in high-throughput data that are highly correlated with sample phenotypes to reveal patterns of gene association among different samples and to detect candidate biomarkers.^[23]^ In this study, the R package WGCNA was used to analyze the DEGs and screen gene modules related to immune cells for subsequent analysis. Immune cell analysis showed high proportion of macrophages, neutrophils, and monocytes in the samples, thus genes related to 3 immune cells were selected.

### 2.5. Functional enrichment analysis

To explore the biological function of genes, the R package “clusterProfile” (version 4.0.5) was used to perform Gene Ontology (GO) function and Kyoto Encyclopedia of Genes and Genomes (KEGG) pathway analysis of the module genes.^[24]^ *P* value < .05 were set as the threshold for significant differences, and top 10 entries were selected for presentation.

### 2.6. Identification of immune cell-associated mitochondrial genes

A total of 1136 mitochondrial genes were downloaded from the MitoCarta 3.0 database,^[25]^ and then analyzed by Venn analysis with the above WGCNA module genes using an online tool (https://bioinfogp.cnb.csic.es/tools/venny/). The shared genes were obtained as immune cell-related mitochondrial genes for further analysis.

### 2.7. Protein–protein interaction (PPI) analysis

To explore the interactions between proteins produced by mitochondrial genes, STRING (version: 11.0, http://string-db.org/) was used to establish a PPI network. Subsequently, the top 30 important genes in the network were predicted using 5 topology analysis algorithms (Maximum Neighborhood Component, Maximal Clique Centrality, Edge Percolated Component, Degree, and Deep Neural Network for Clustering) in the cytoHubba plug-in of Cytoscape (Version: 3.8.2).^[26]^

### 2.8. Identification of diagnostic genes by machine learning

Three machine learning algorithms, including least absolute shrinkage and selection operator (LASSO), random forest (RF), and Support Vector Machine-Recursive Feature Elimination (SVM-RFE), were used to screen the potential biomarkers for ADRS diagnosis. LASSO used the LI-penalty parameter (lambda) to set the coefficients of insignificant variables to zero, and selected characteristic variables as candidate biomarkers by 10-fold cross-validation.^[27]^ RF method was validated by 10-fold cross-validation to build a RF model, and genes with MeanDecreaseGini > 4 were selected as important variables. In addition, recursive feature elimination in SVM-RFE was applied to rank the genes and determine the feature genes. Three methods were conducted via glmnet (version 4.1-3),^[28]^ randomForest (version 4.6-14),^[29]^ and e1071 (version 1.7-9),^[30]^ respectively. The interaction genes of the 3 algorithms were considered as the diagnostic biomarkers of ARDS. Furthermore, multivariate logistic regression was used to calculate the regression coefficient and expression level of each gene to establish the Score formula, as follows: Score = β_1_X_1_ + β_2_X_2_ + ... +β_n_X_n_. Here, β is the regression coefficient, and X is the expression value of gene.

### 2.9. Validation and performance evaluation of the diagnostic score

According to the formula, the score value of each sample in the training and validation sets were calculated, and then differences in score between ARDS and sepsis were compared by Wilcoxon test. Moreover, R language pROC (version 1.7.2)^[31]^ was utilized to plot the receiver operating characteristic (ROC) and calculate the area under the curve (AUC) to evaluate the accuracy of score in all datasets.

### 2.10. Establishment of nomogram model

First, Pearson was applied to analyze the correlation between diagnostic genes and clinical information. Next, rms package (version 5.1-2) was used to integrate all diagnostic genes to construct a nomogram model for ARDS diagnosis. The decision curve and clinical impact curve were employed to reveal the clinical value of model.

### 2.11. Correlation analysis of diagnostic genes and immune cells

Based on the training set, ssGSEA and CIBERSORT algorithms were used to calculate the proportion of immune cells in samples. Correlation of diagnostic genes and immune cells were calculated using Pearson.

### 2.12. PPI and GSEA assay

PPI analysis of diagnostic genes and their 20 interacting genes were performed using the GeneMANIA database (http://genemania.org/). In addition, GSEA was used to analyze the significant KEGG enrichment between ARDS and sepsis. Among them, *P* value < .05 and |NES| > 1 were set as cutoff threshold. Meanwhile, KEGG pathways were scored using the GSVA algorithm, and then association between diagnostic genes and pathway scores were calculated via Pearson.

### 2.13. Prediction of drugs for diagnostic genes

Drug Signatures Database (https://dsigdb.tanlab.org/DSigDBv1.0/) is a collection that links drugs/compounds and their target genes.^[32]^ In this study, the relationship between diagnostic genes and drugs was analyzed by Drug Signatures Database, and the results were visualized by Cytoscape, only showing Down drugs for each gene.

### 2.14. Clinical samples collection

Patients diagnosed with sepsis at our hospital were included in this study. Inclusion criteria were as follows: (1) age ≥ 18; (2) hospitalization time > 24 hours; (3) patients met the diagnostic criteria of sepsis 3.0.^[33]^ Exclusion criteria applied to patients who (1) were discharged or death within 24 hours of admission, (2) underwent emergency surgery after admission, and (3) had malignant tumors and were pregnant or breastfeeding. Patients were classified as having ARDS if they presented with acute hypoxemia (partial pressure of arterial oxygen/fraction of inspired oxygen ≤ 300), bilateral pulmonary infiltrates on chest radiography, and no clinical evidence of left atrial hypertension.^[34]^ Finally, 5 patients with sepsis and 5 patients with ARDS were included, followed by collection of blood samples. This study was approved by the Ethics Committee of Jiangnan University Medical Center and obtained the informed consent from each case.

### 2.15. Experimental validation

Total RNA was extracted from blood samples using TRIzol regent and assayed for concentration and quality. Following the manufacturer’s instructions, 500 ng of total RNA was reverse transcribed to complementary DNA and then subjected to real-time reverse transcriptase-polymerase chain reaction (RT-qPCR) on the ABI 7500 Fast Real-time PCR system using the TB Green Premix Ex Taq II kit. The relative expression of diagnostic genes was calculated using the 2^-△△CT^ method, with glyceraldehyde 3-phosphate dehydrogenase as an internal reference.

### 2.16. Statistical analysis

All data were analyzed using R software (version 4.1.3) and GraphPad (version 8.0). Comparisons between the 2 groups were evaluated using the Wilcoxon test and multiple testing was corrected by the Benjamini–Hochberg method. Moreover, correlations between variables were assessed using Pearson. A statistically significant difference was defined as *P* value < .05.

## 3. Results

### 3.1. ARDS and sepsis samples had different immune cell profiles

**Figure 2.**
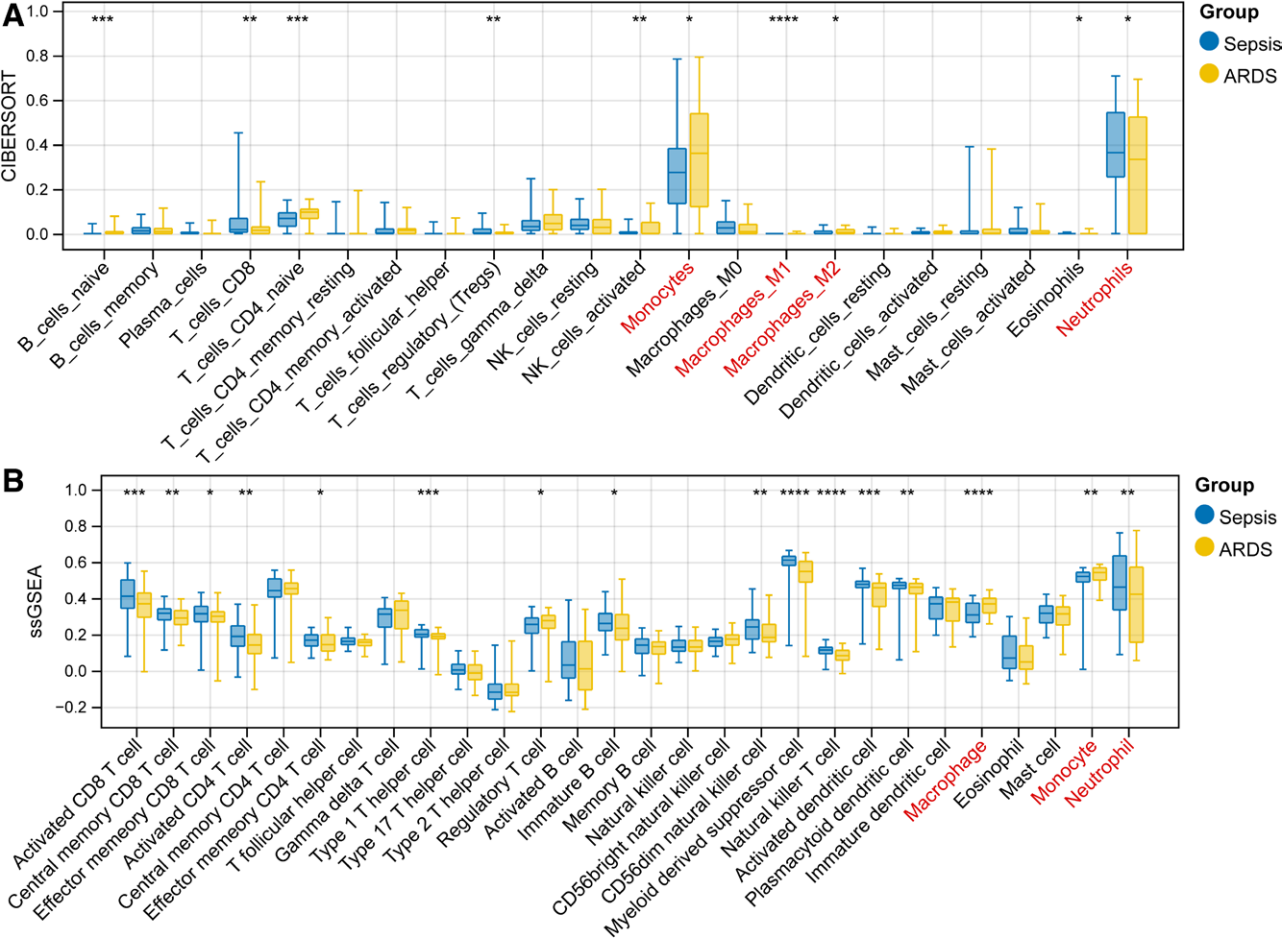
Violin plot showing the percentage infiltration of immune cells in ARDS and sepsis. (A) Immune infiltration analysis of 22 immune cells by using CIBERSORT. (B) Immune infiltration analysis of 28 immune cells by ssGSEA. *P < .05, **P < .01, ***P < .001, and ****P < .0001 (Wilcoxon test, ARDS vs sepsis). ARDS = acute respiratory distress syndrome, ssGSEA = single-sample gene set enrichment analysis.

Three Gene Expression Omnibus datasets (training set) were subjected to bath effect removal and merged, resulting in 86 ARDS and 86 sepsis samples for analysis. CIBERSORT algorithm obtained the proportion of 22 immune cells in each sample, and 10 differential immune cells (DICs) were found between ARDS and sepsis (Fig. 2A). In addition, ssGSEA algorithm obtained 16 DICs in 2 groups (Fig. 2B). Notably, 2 algorithms found that macrophages, neutrophils, and monocytes were DICs, with higher degree of infiltration in the samples. Compared to sepsis, macrophages and monocytes were observably increased in ARDS, whereas neutrophils were decreased. Hence, these immune cells were selected as key cells for further analysis.

### 3.2. Screening for DEGs between ARDS and sepsis

Based on set thresholds, 1049 DEGs were screened between ARDS and sepsis, including 1037 up- and 12 down-regulated genes (Figure S1A, Supplemental Digital Content, http://links.lww.com/MD/O416). Of which, top 50 DEGs were displayed using Heatmap (Figure S1B, Supplemental Digital Content, http://links.lww.com/MD/O416).

### 3.3. Immune cells-associated key modules identified by WGCNA

**Figure 3.**
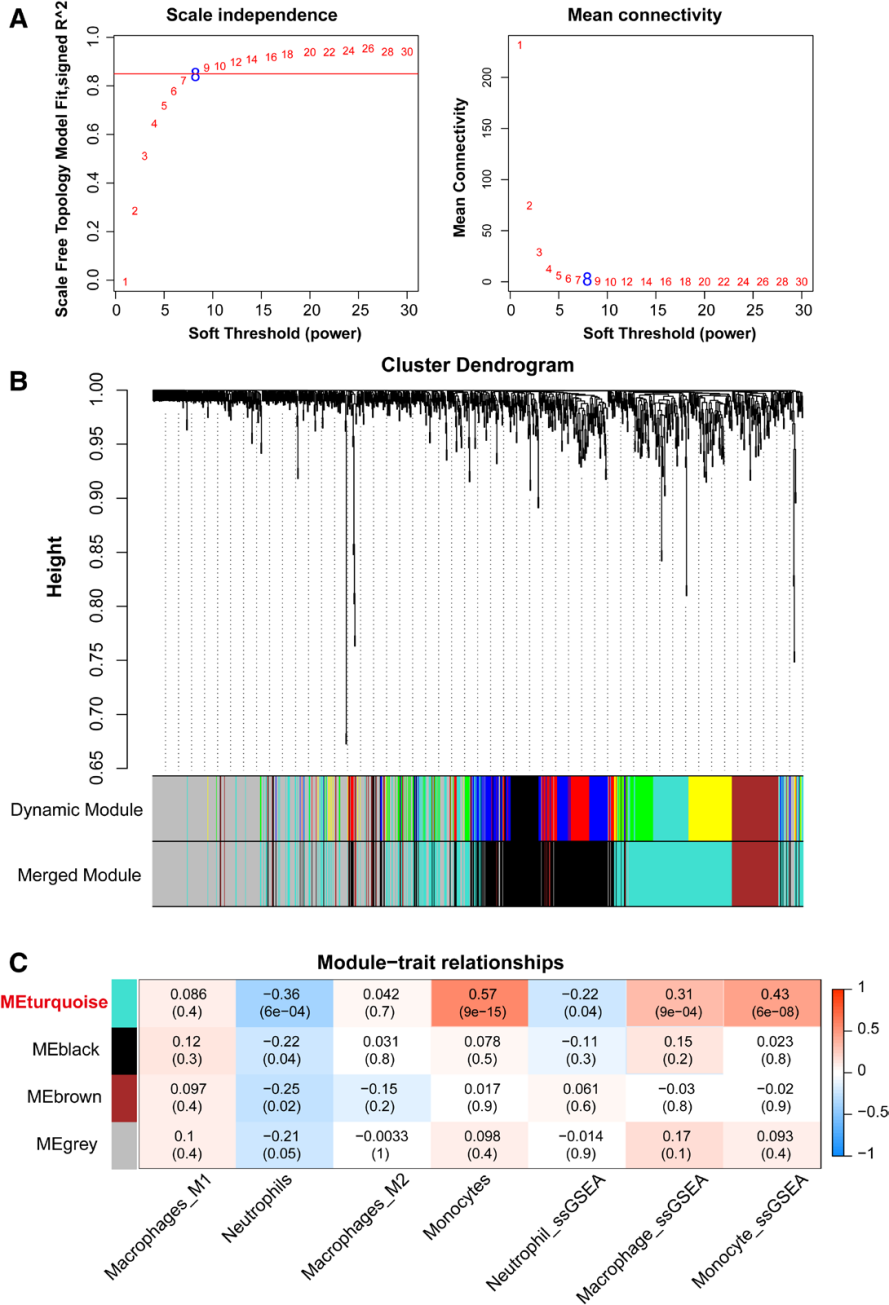
Screening of module genes related to immune cells by WGCNA. (A) Determine the weight parameter (power) that satisfies the scale-free network distribution. Red line indicates the standard line where the R2 of correlation coefficient reaches 0.85. (B) Tree diagram of the partition of modules. Each color refers to a different module. (C) Heatmap of the correlation between each module and immune cell phenotype. WGCNA = weighted gene co-expression network analysis.

Based on the expression levels of DEGs, WGCNA analysis was performed with immune scores for macrophages, neutrophils, and monocytes as phenotypes. As shown in Figure 3A, when the *R*^2^ value reached 0.85 (red line, power = 8), the scale-free network distribution was satisfied, and the average connectivity value of the adjacency matrix was relatively high. Next, 4 distinct co-expression modules were identified by clustering and dynamic tree cutting method (Fig. 3B). In addition, the correlation between modules and phenotypes was analyzed and turquoise module was found to show the highest correlation with immune cells (Fig. 3C). Therefore, 298 genes in this model were selected for further analysis.

### 3.4. GO terms and KEGG pathways of 298 module genes

Functional enrichment analysis was performed to reveal the biological function of module genes. For GO results, genes were mainly enriched in lipid oxidation (GO_Biological process), unfold protein binding (GO_Molecular function), and mitochondrial matrix (GO_Cellular components) (Figure S2A–4C, Supplemental Digital Content, http://links.lww.com/MD/O416). In terms to KEGG, genes were mainly involved in lysosome, carbon metabolism, and pyruvate metabolism (Figure S2D, Supplemental Digital Content, http://links.lww.com/MD/O416).

### 3.5. PPI network of immune cells-related mitochondrial genes

**Figure 4.**
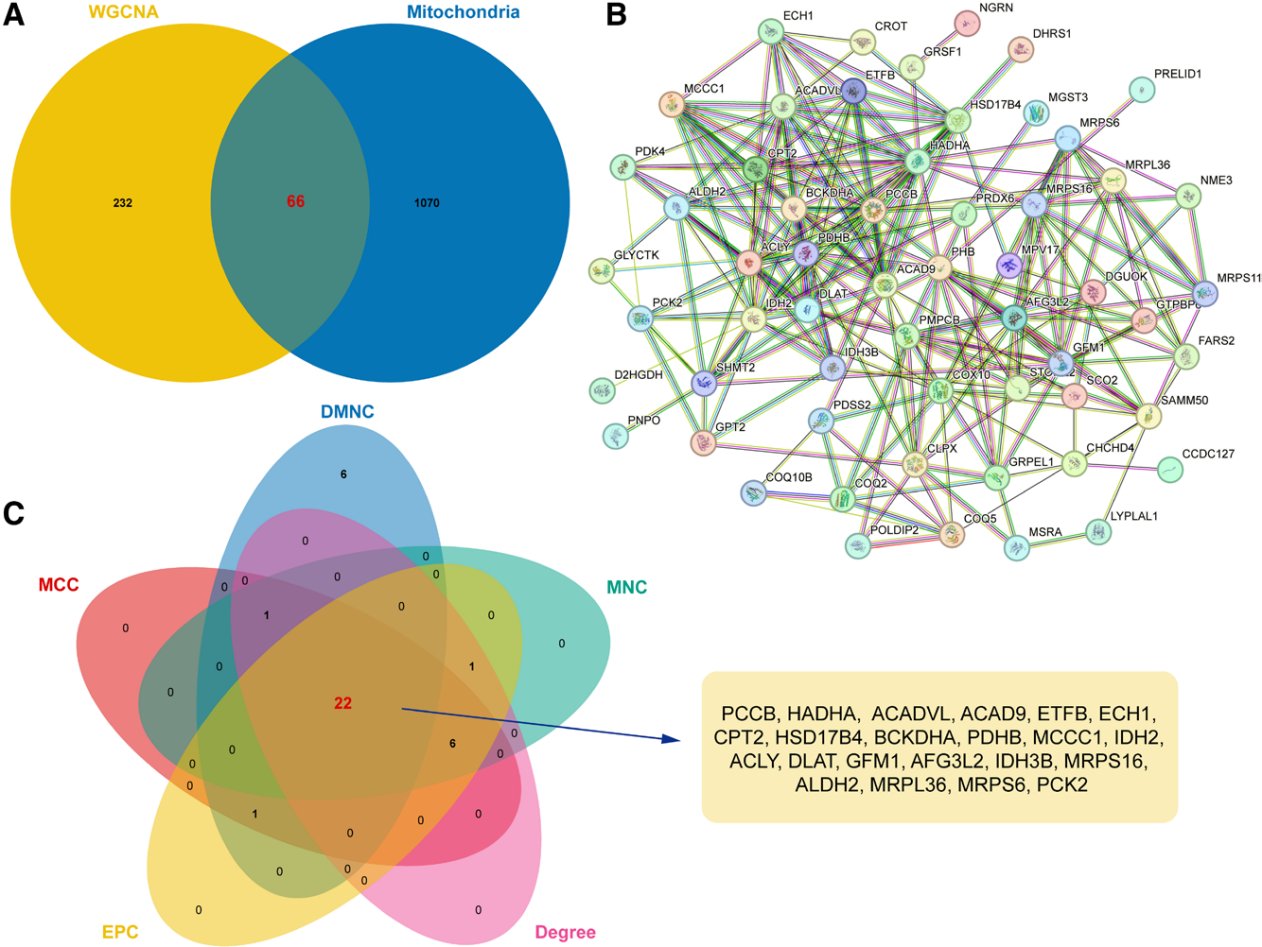
PPI analysis of immune cells-related mitochondrial genes. (A) Integrated analysis of mitochondrial genes and module genes. (B) PPI network of common genes. (C) Venn diagram showing the key nodes in PPI network revealed by 5 different topology analysis algorithms. PPI = protein–protein interaction.

After the integration of module genes and mitochondrial genes, 66 shared genes were obtained as immune cells-related mitochondrial genes (Fig. 4A). Based on these genes, a PPI network containing 46 nodes and 236 edges were established (Fig. 4B). Next, hub genes in PPI network were screened via 5 topology analysis algorithms, and 22 common genes were selected, such as Propionyl-CoA carboxylase subunit beta (PCCB), Hydroxyl CoA dehydrogenase alpha subunit, and Acyl-CoA dehydrogenase very long chain (ACADVL) (Fig. 4C).

### 3.6. Five diagnostic biomarkers were identified via 3 machine learning algorithms

**Figure 5.**
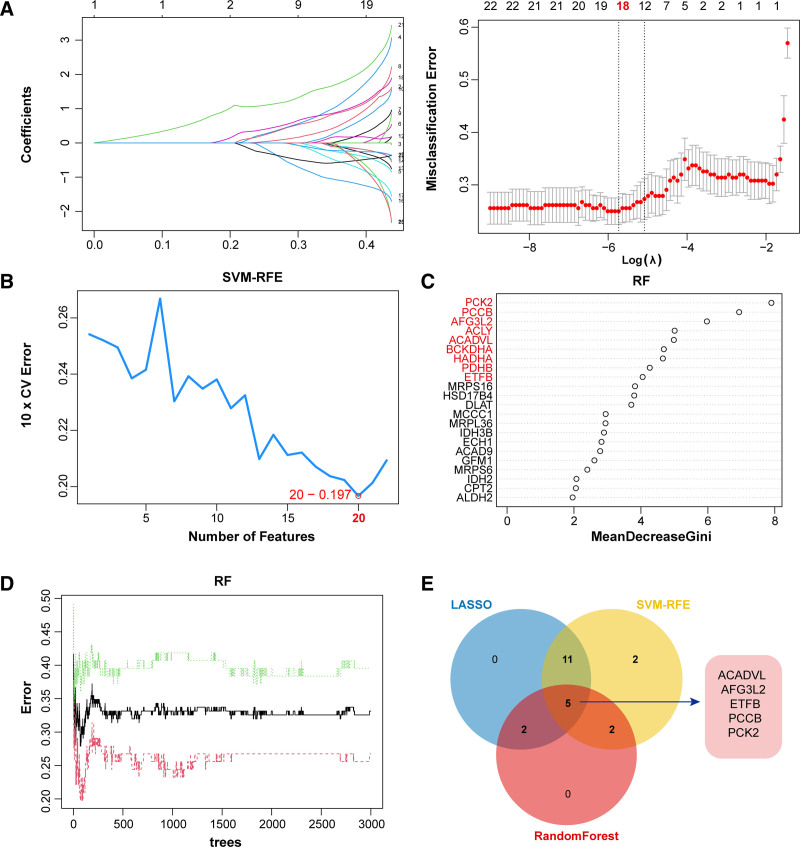
Identification of biomarkers for ARDS via 3 machine learning algorithms. (A) LASSO regression model selecting the optimal parameters (λ = 18) through cross-validated ten times. (B) SVM-RFE model showing the minimum error value when the number of features is 20. (C and D) RF algorithm revealing 9 important variables based on MeanDecreaseGini > 4. (E) Venn diagram exhibiting the potential biomarkers among 3 algorithms. ARDS = acute respiratory distress syndrome, LASSO = least absolute shrinkage and selection operator, RF = random forest, SVM-RFE = Support Vector Machine-Recursive Feature Elimination.

Three algorithms were applied to select biomarkers among 22 above genes. The LASSO classifier identified 18 signature genes (Fig. 5A). The SVM-RFE algorithm showed the minimum classifier error when the number of feature genes were 20 (Fig. 5B). Moreover, the RF model determined 9 genes based on MeanDecreaseGini > 4 (Fig. 5C and D). Finally, 5 overlapping genes were obtained as diagnostic genes of ARDS by Venn analysis, including ACADVL, ATPase family gene 3-like 2 (AFG3L2), electron transfer flavoprotein subunit beta (ETFB), PCCB, and phosphoenolpyruvate carboxykinase 2 (PCK2) (Fig. 5E).

### 3.7. Calculation of diagnostic score and assessment of predictive performance

**Figure 6.**
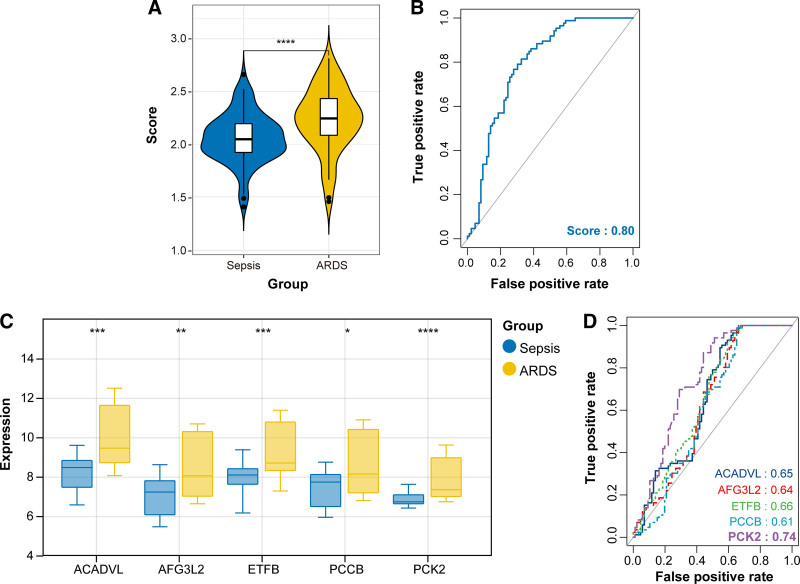
Assessment of predictive performance for 5 diagnostic genes in the training set. (A) Diagnostic score between sepsis and ARDS groups. (B) ROC curve for combined 5 genes for ARDS diagnosis. (C) mRNA expression level of 5 genes in sepsis and ARDS samples. (D) ROC curve of each gene for ARDS diagnosis. ARDS = acute respiratory distress syndrome, ROC = receiver operating characteristic.

**Figure 7.**
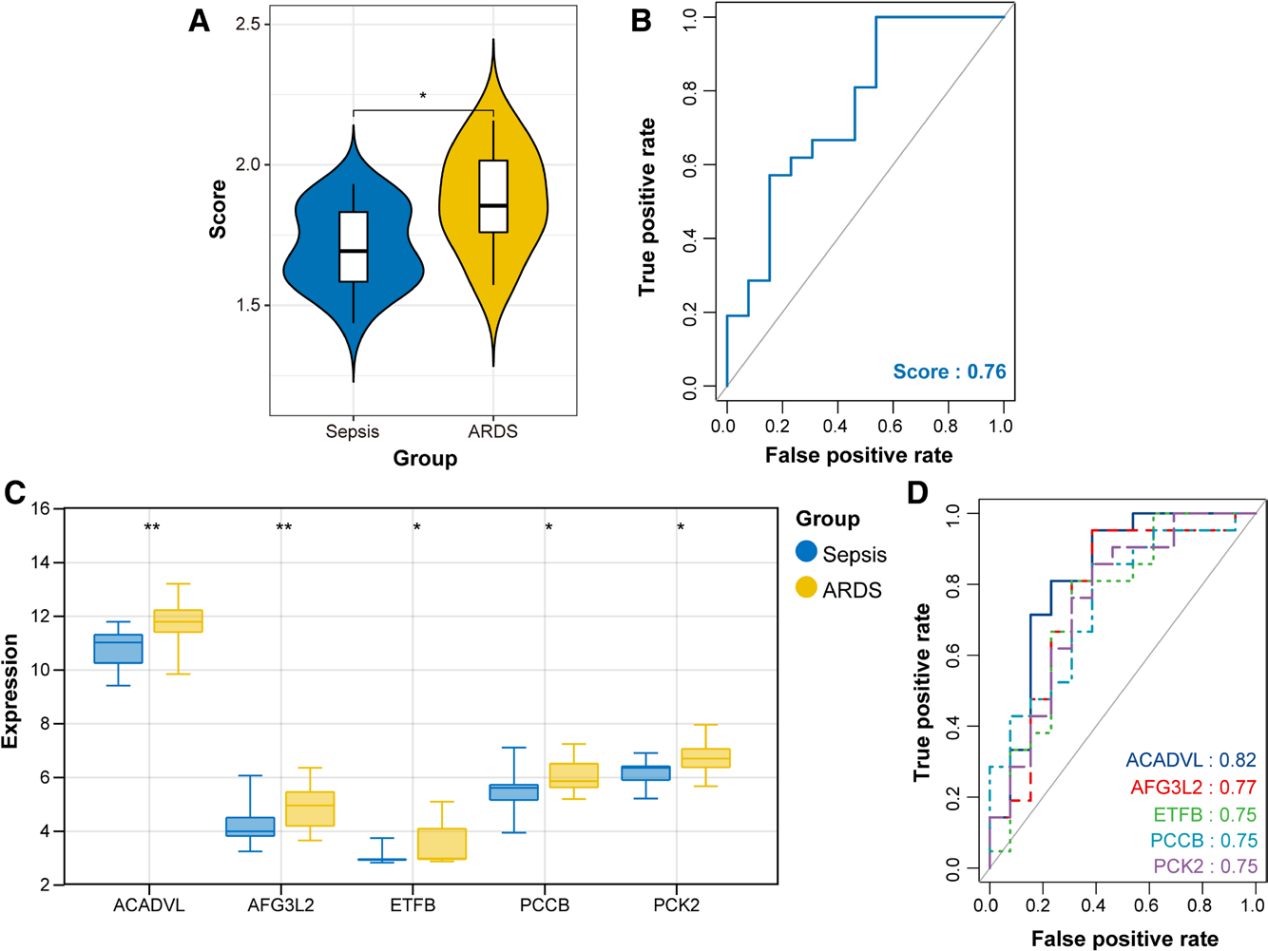
Calculation of predictive performance for 5 diagnostic genes in the validation set. (A) Diagnostic score between sepsis and ARDS groups. (B) ROC curve for combined 5 genes for ARDS diagnosis. (C) mRNA expression level of 5 genes in sepsis and ARDS samples. (D) ROC curve of each gene for ARDS diagnosis. *P < .05; **P < .01; ***P < .001; ****P < .0001. ARDS = acute respiratory distress syndrome, ROC = receiver operating characteristic.

Diagnostic scores were calculated based on the regression coefficient (multivariate logistic analysis) and expression levels of 5 diagnostic genes, with the following formula: Score = ACADVL × 0.0523 + AFG3L2 × 0.1162 - ETFB × 0.0087 - PCCB × 0.1314 + PCK2 × 0.2274. Using this formula, diagnostic score was estimated for each sample in the training and validation sets. It was found that the diagnostic score was able to distinguish ARDS from sepsis samples, where the score was significantly higher in ARDS than in sepsis patients (Fig. 6A). ROC curve showed an AUC value of 0.80 for this score, indicating that is had a good performance in separating ARDS from sepsis (Fig. 6B). Result showed that the expression levels of 5 genes were markedly different between ARDS and sepsis, especially exhibiting high expression in ARDS samples (Fig. 6C). We also evaluated the predictive performance of each gene and found that PCK2 had a relatively high AUC value (0.74) (Fig. 6D). Similar results were observed in the validation set. Specifically, the diagnostic score was significantly higher in the ARDS sample compared to the sepsis sample, with an AUC of 0.76 (Fig. 7A and B). All 5 genes were up-regulated in ARDS versus sepsis samples (Fig. 7C). The ROC curves showed that genes had good discrimination efficiency, and the AUC of all genes was >0.75 (Fig. 7D). Together, these results indicated that the diagnostic model had satisfactory predictive performance.

### 3.8. Construction of a diagnostic nomogram model containing 5 genes

**Figure 8.**
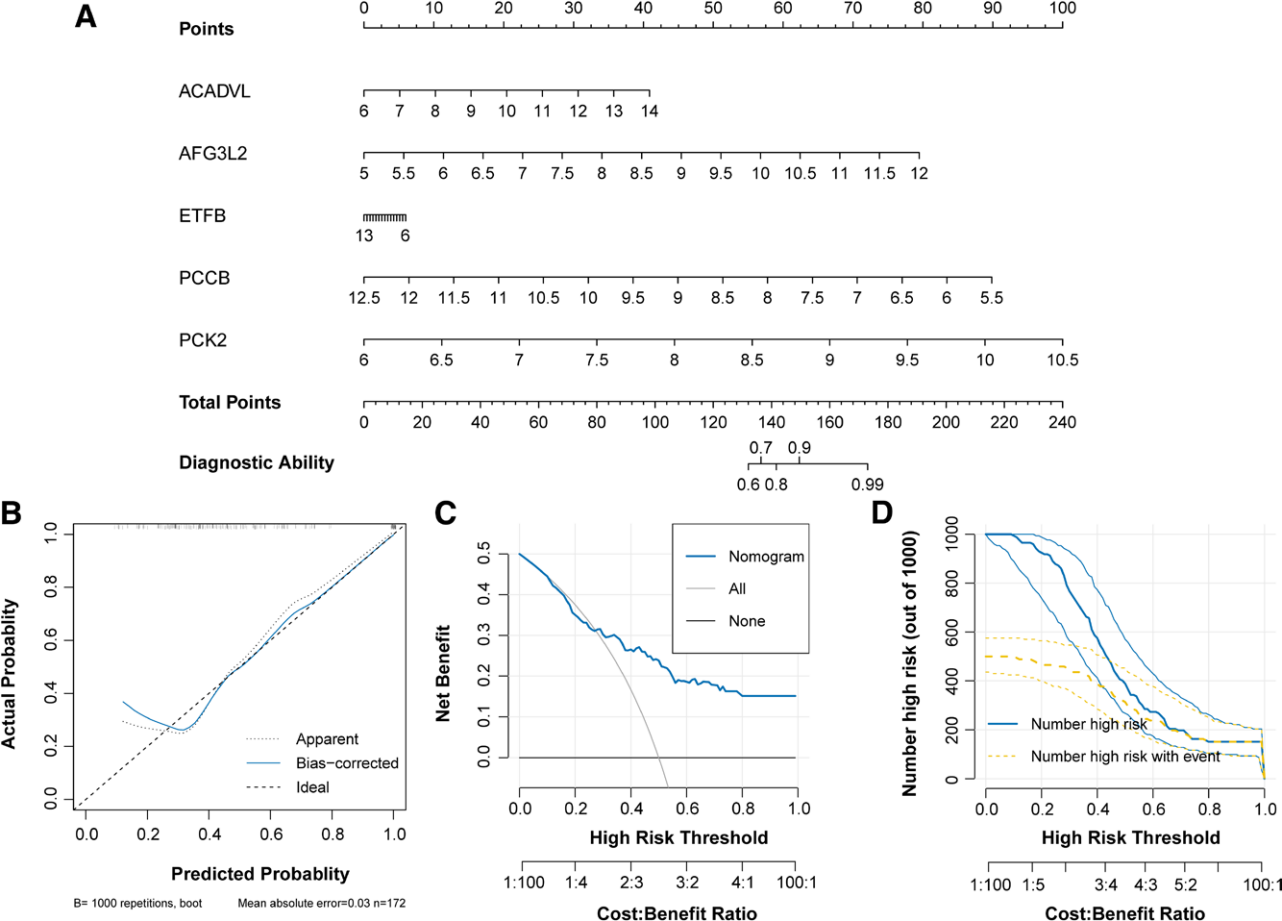
Establishment and evaluation of nomogram model. (A) Nomogram model composing of 5 diagnostic genes. (B) Calibration curve reveling the agreement of actual risk and predicted risk. (C) Decision curve and (D) clinical impact curve showing the clinical application value of model.

Relationship between genes and clinical features was analyzed. Most of the genes showed significant negative correlation with closest_ant and WBC (Figure S3A, Supplemental Digital Content, http://links.lww.com/MD/O416). In addition, the expression level of ACADVL was associated with aecc_ali, bath_2, and berlin_ards (Figure S3B–3D, Supplemental Digital Content, http://links.lww.com/MD/O416 f). Subsequently, we incorporated these 5 genes into the nomogram model and evaluated their diagnostic ability for ARDS. Results showed that PCK2 contributed the most to the outcome events, followed by PCCB and AFG3L2 (Fig. 8A). The calibration curve revealed that the actual risk of ARDS was consistent with the predicted risk, indicating that the nomogram had accurate predictive power (Fig. 8B). In the decision curve analysis, the curve of the monogram was higher than the gray curve, suggesting that clinical patients could benefit from the nomogram model (Fig. 8C). The clinical impact curve further confirmed that patients achieved greater clinical benefit at a high-risk threshold of 0.7 to 1.0 (Fig. 8D). Overall, these results indicated that the nomogram had ideal predictive performance.

### 3.9. Correlation between diagnostic genes and immune cells

**Figure 9.**
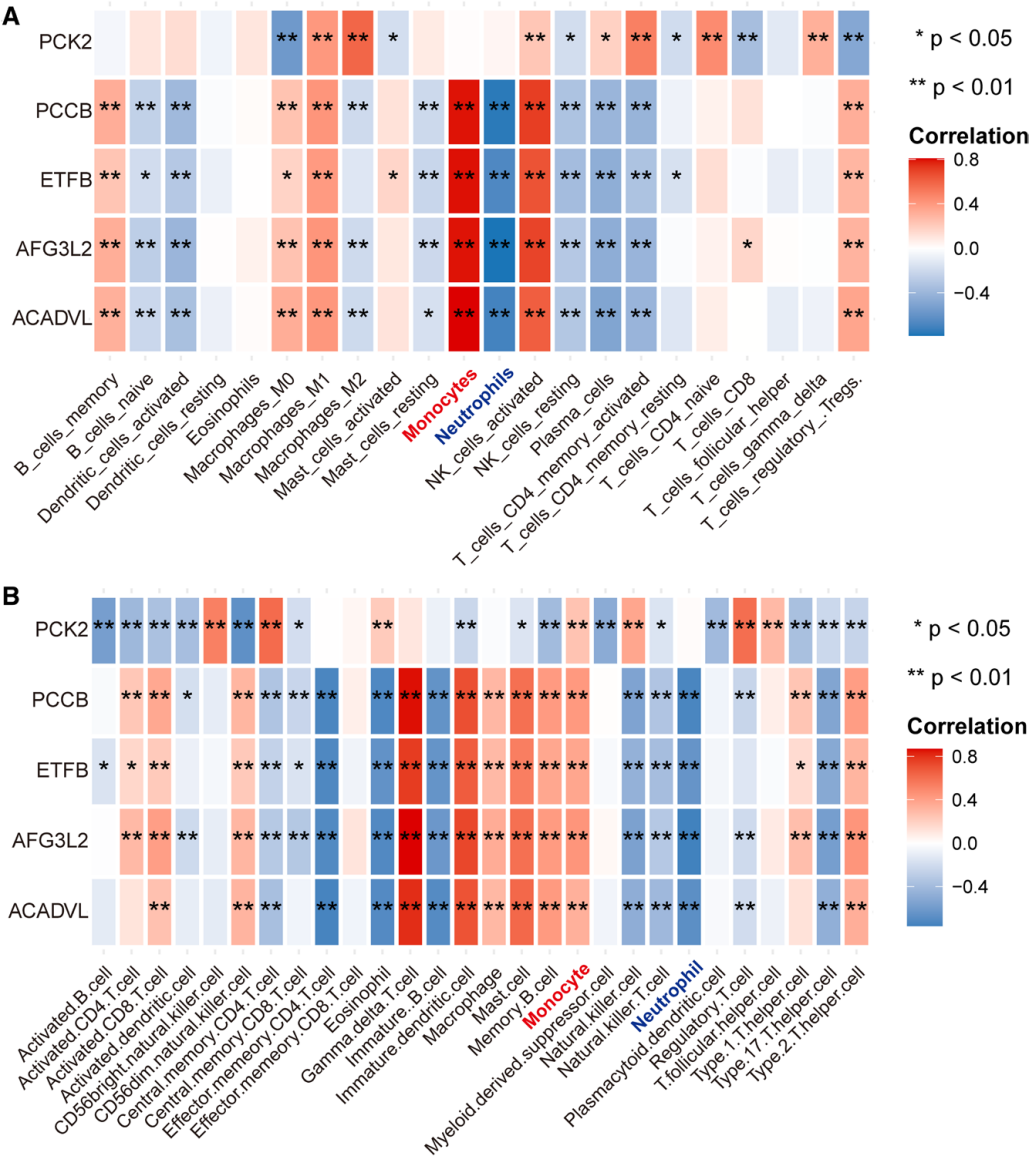
Correlation between diagnostic genes and immune cells based on (A) CIBERSORT algorithm and (B) ssGSEA algorithm. *P < .05; **P < .01. ssGSEA = single-sample gene set enrichment analysis.

We assessed the relationship between diagnostic genes and immune cells, focusing on 3 immune cells obtained previously. As shown in Figure 9A and B, all diagnostic genes showed a strong positive correlation with monocytes, while exhibited varying degrees of negative association with neutrophils.

### 3.10. PPI, KEGG pathway analysis, and drug prediction of diagnostic genes

**Figure 10.**
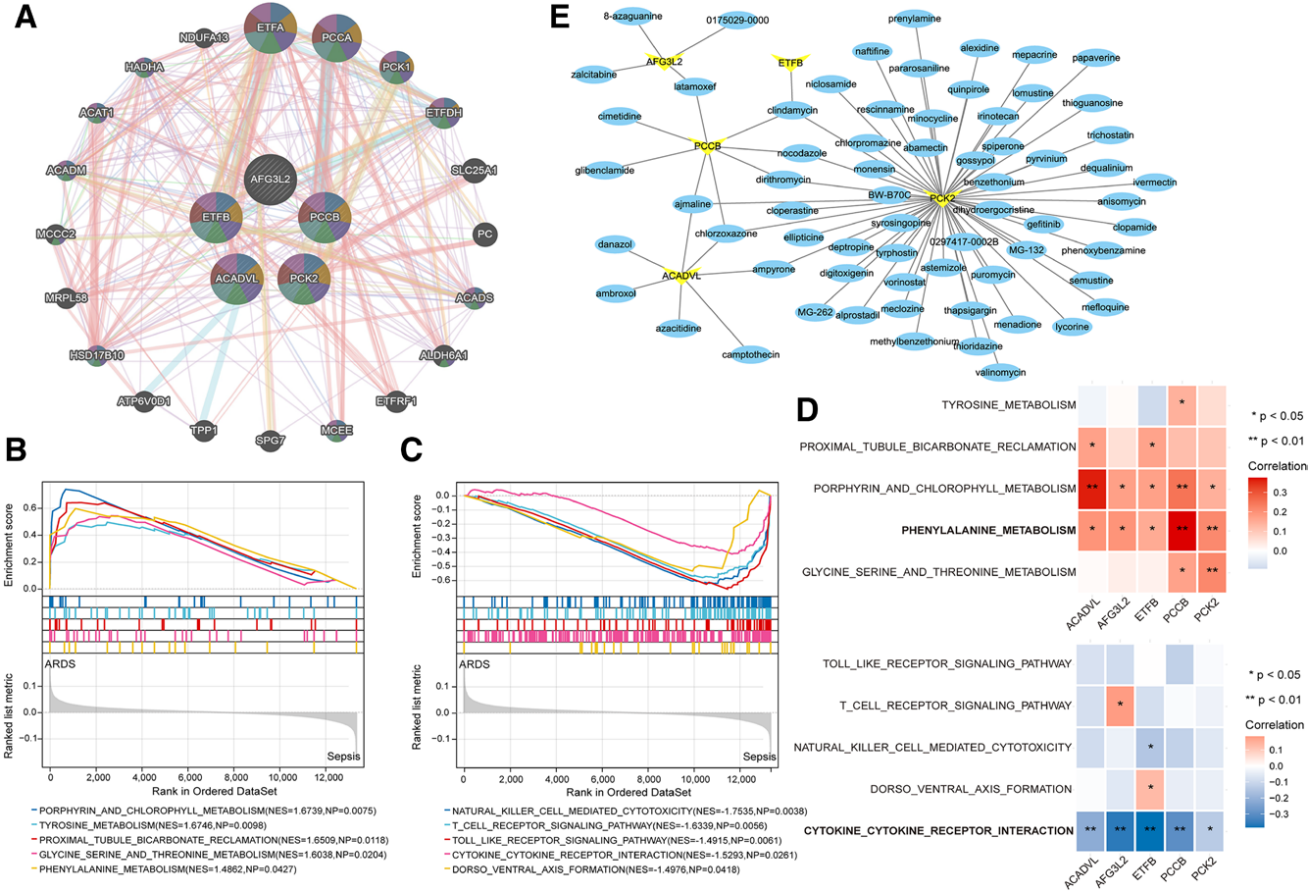
GSEA and drug prediction of diagnostic genes. (A) PPI network of diagnostic genes and their significantly related 20 genes. (B and C) GSEA revealing the top 5 pathways involved in sepsis and ARDS. (D) Correlation between diagnostic genes and KEGG pathways. (E) Diagnostic gene–drug network. Yellow node represents gene and blue node represents drug. *P < .05; **P < .01. ARDS = acute respiratory distress syndrome, GSEA = gene set enrichment analysis, KEGG = Kyoto Encyclopedia of Genes and Genomes, PPI = protein–protein interaction.

PPI network of the diagnostic genes and their significantly related 20 genes were constructed using the GeneMANIA database (Fig. 10A). The signaling pathways involved in the diagnostic genes were also explored. The expression of genes in the ARDS group was increased in the following pathways: porphyrin and chlorophyl metabolism, tyrosine metabolism, proximal tubule bicarbonate reclamation, glycine serine and threonine metabolism, and phenylalanine (Fig. 10B). Genes in the sepsis group were mainly involved in natural killer cell mediated cytotoxicity, T cell receptor signaling pathway, toll like receptor signaling pathway, cytokine–cytokine receptor interaction, and dorso-ventral axis formation (Fig. 10C). Notably, correlation analysis showed that the diagnostic genes mainly presented a positive correlation with phenylalanine metabolism, while a negative correlation with cytokine–cytokine receptor interaction (Fig. 10D). Furthermore, drugs interacting with the 5 feature genes were predicted. As shown in Figure 10E, 3 drugs including chlorzoxazone, ajmaline, and clindamycin were closely linked to multiple genes.

### 3.11. Validation of expression of biomarkers by RT-qPCR

**Figure 11.**
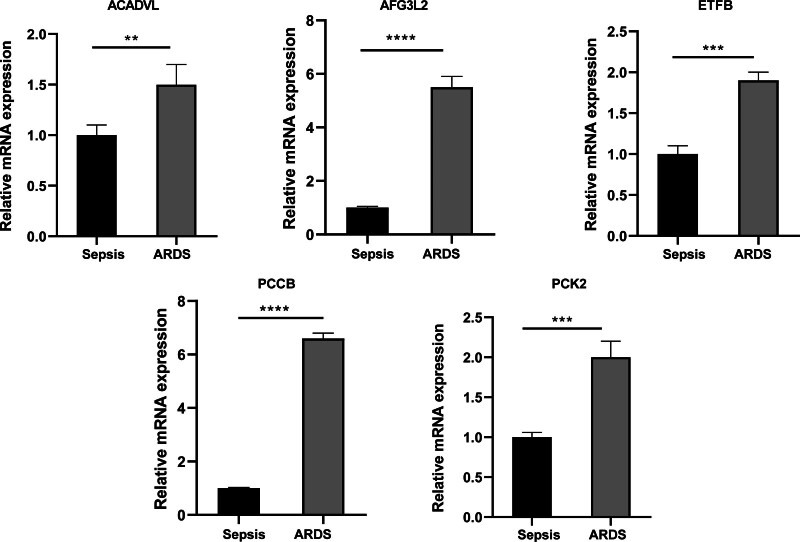
mRNA expressions of biomarkers detected by RT-qPCR assay in sepsis and ARDS. **P < .01; ***P < .001; ****P < .0001. RT-qPCR = real-time reverse transcriptase-polymerase chain reaction.

To verify the expression pattern of key mitochondrial genes in ARDS patients, RT-qPCR assay was performed. Our team found that the expression levels of ACADVL, AFG3L2, ETFB, PCCB, and PCK2 were significantly overexpressed in ARDS samples compared to sepsis samples (all *P* value < .05, Fig. 11), which was consistent with the results of bioinformatics analysis.

## 4. Discussion

Due to the complexity of ARDS pathogenesis and the heterogeneity of the patient population, the development of specific therapeutic measures for ARDS is a great challenge. There is growing evidence that the identification of biomarkers contributes to a better understanding of the pathobiology of ARDS and can be utilized for risk stratification of subjects in clinical trials.^[35,36]^ Despite the gap between biomarker discovery and effective translation into clinical applications, the identification novel markers and combinatorial model construction may hold promise for the exploitation of targeted therapies.^[37]^

In this study, we first analyzed the differences in immune cell infiltration in ARDS and sepsis. Results showed that compared to sepsis, the proportion of macrophages and monocytes was higher in ARDS, while neutrophils were lower. Macrophages play a dual role in the inflammatory process of ARDS, mainly depending on its polarized phenotype.^[38]^ In the early phase, alveolar macrophages convert to the M1 phenotype to release various pro-inflammatory mediators. In the late stage, the activated M1 phenotype switches to the M2 phenotype to remove apoptotic cells and reduce inflammation.^[39]^ However, over-polarization of M2 may cause pulmonary fibrosis.^[40]^ Besides, in patients with ARDS, peripheral blood monocytes are recruited into the alveolar lumen and then differentiate into M1 macrophages, which is responsible for the poor survival of patients.^[39]^ In addition, the degree of neutrophil apoptosis in peripheral blood is inversely proportional to the severity of sepsis-induced ARDS, which is consistent with our observation.^[41]^ These studies emphasize the potential of modulating macrophages, monocytes, and neutrophils in the treatment of ARDS.

Following, we used WGCNA to explore the module genes closely associated with the 3 immune cell phenotypes. Functional enrichment analysis revealed that these genes were enriched in mitochondria-related terms, such as mitochondrial matrix. Hence, by integrating module and mitochondrial genes, we finally obtained 5 key immune-related diagnostic genes (ACADVL, AFG3L2, ETFB, PCCB, and PCK2) via bioinformatics analysis and machine learning. ACADVL is recognized as key mediator of mitochondrial energy metabolism, especially fatty acid metabolism.^[42]^ There is evidence that abnormal increases in fatty acid oxidation-related proteins, including ACADVL, disrupt mitochondrial energy metabolism in the lung of piglets and participate in the process of pulmonary fibrosis.^[43]^ The protein encoded by AFG3L2 constitutes the mitochondrial m-AAA protease, which is a core part involved in mitochondrial quality control.^[44]^ Studies have implicated that AFG3L2 regulates respiratory chain integrity and mitochondrial structure; meanwhile, it also indirectly affects mtDNA release and inflammatory response.^[45,46]^ ETFB functions as an electron receptor between mitochondrial fatty acid catabolism and ATP production.^[47]^ ETFB instability has been observed in cases of cataract, alopecia, oral mucosal disorder, and psoriasis-like syndrome, leading to severe inflammatory lesions by impairing mitochondrial respiration and reducing β-oxidative activity.^[48]^ PCCB is also related to abnormal fatty acid metabolism and has been confirmed to be involved in the pathogenesis of propionic acidemia.^[49]^ PCK2 serves an important role in the regulation of mitochondrial respiration and redox balance.^[50]^ In addition, PCK2 overexpression accelerates lipopolysaccharide-induced inflammatory responses, whereas gene knockdown inhibits the activation of the inflammatory cascade response.^[51]^ Altogether, these genes can affect mitochondrial metabolism-related biological functions, but their roles in the pathogenesis of ARDS are still unclear. Moreover, we collected samples from sepsis and ARDS, and confirmed the expression of the diagnostic genes by RT-qPCR, proving for the first time the potential role of these genes in the ARDS pathogenesis.

Based on the training and validation sets, we analyzed the expression levels and diagnostic capabilities of these genes. All genes were found to be up-regulated in ARDS compared with sepsis. As expected, these genes had certain diagnosis value in ARDS. Notably, the nomogram composed of 5 genes also had better diagnostic efficacy than the single gene in patients with ARDS. Besides, the nomogram showed ideal predictive ability and had clinical application value. Hence, we speculate that this nomogram can significantly distinguish between ARDS and sepsis.

In this analysis, we also explore the biological function and targeted drugs of genes. Diagnostic genes exhibited a positive correlation with phenylalanine metabolism. Phenylalanine is known to be an essential amino acid that enhances immune response and inflammation.^[52]^ Recent study has indicated that phenylalanine promotes alveolar macrophage pyroptosis and release pro-inflammatory factors (IL-1β and IL-18), which aggravates lung injury and ARDS death in mice, suggesting phenylalanine may be a promising target for ARDS therapy.^[53]^ In addition, drug prediction revealed strong associations of chlorzoxazone, ajmaline, and clindamycin with multiple diagnostic genes. Among them, clindamycin exerts antibacterial role and inhibit toxins, which can reduce the mortality of patients with streptococcal toxic shock syndrome. Unfortunately, the effects of these agents on biomarkers and ARDS treatment have not been reported.

To our knowledge, this study is the first to screen diagnostic biomarkers for ARDS from the perspective of the combined immune cells and mitochondrial genes, and the constructed nomogram had good application value in predicting the occurrence of ARDS in patients with sepsis. However, our research has certain limitations. First, the role of diagnostic genes in the pathogenesis of ARDS is lack of literature support, and cell/animal experiments are needed to reveal their specific functions. Second, the predictive model is validated only in a dataset with a small sample size, and future large-scale cohorts are required to confirm the feasibility of our findings.

## 5. Conclusion

In summary, we presented a comprehensive analysis of sepsis and ARDS based on gene expression profiles and mitochondrial genes. Clear differences in infiltration of macrophages, neutrophils, and monocytes were observed in ARDS and sepsis. Five key ARDS diagnostic genes were identified by WGCNA and machine learning, including ACADVL, AFG3L2, ETFB, PCCB, and PCK2. Then, the expression of these genes was elevated in ARDS compared with sepsis, and the nomogram constructed from 5 genes had good diagnostic ability in ARDS. These findings may provide valuable references for the clinical management of ARDS in the future.

## Author contributions

**Data curation:** Wei Sun, Su Tu.

**Formal analysis:** Wei Sun, Su Tu.

**Writing – original draft:** Wei Sun.

**Writing – review & editing:** Su Tu.

## Supplementary Material
