# Supplementary material for: Screening of mitochondrial-related biomarkers connected with immune infiltration for acute respiratory distress syndrome through WGCNA and machine learning
Source: Medicine (Baltimore). 2025 Mar 7;104(10):e41497. doi: 10.1097/MD.0000000000041497 (PMC11903030; doi:10.1097/MD.0000000000041497)

Figure S1 Identification of DEGs between sepsis and ARDS. (A) Volcano plot representing DEGs. Blue dots indicate down-regulated DEGs and red dots indicate up-regulated DEGs. (B) Heatmap showing top 100 DEGs.

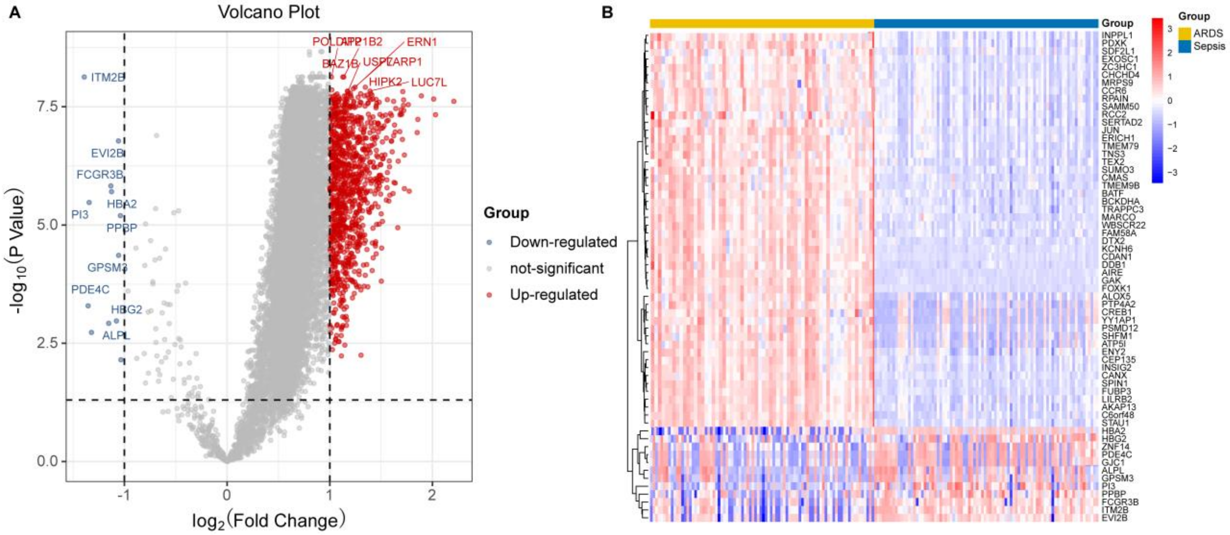

Figure S2 Functional enrichment analysis of module genes. (A) GO biological process analysis. (B) GO molecular function analysis. (C) GO cellular component analysis. (D) Kegg pathway analysis

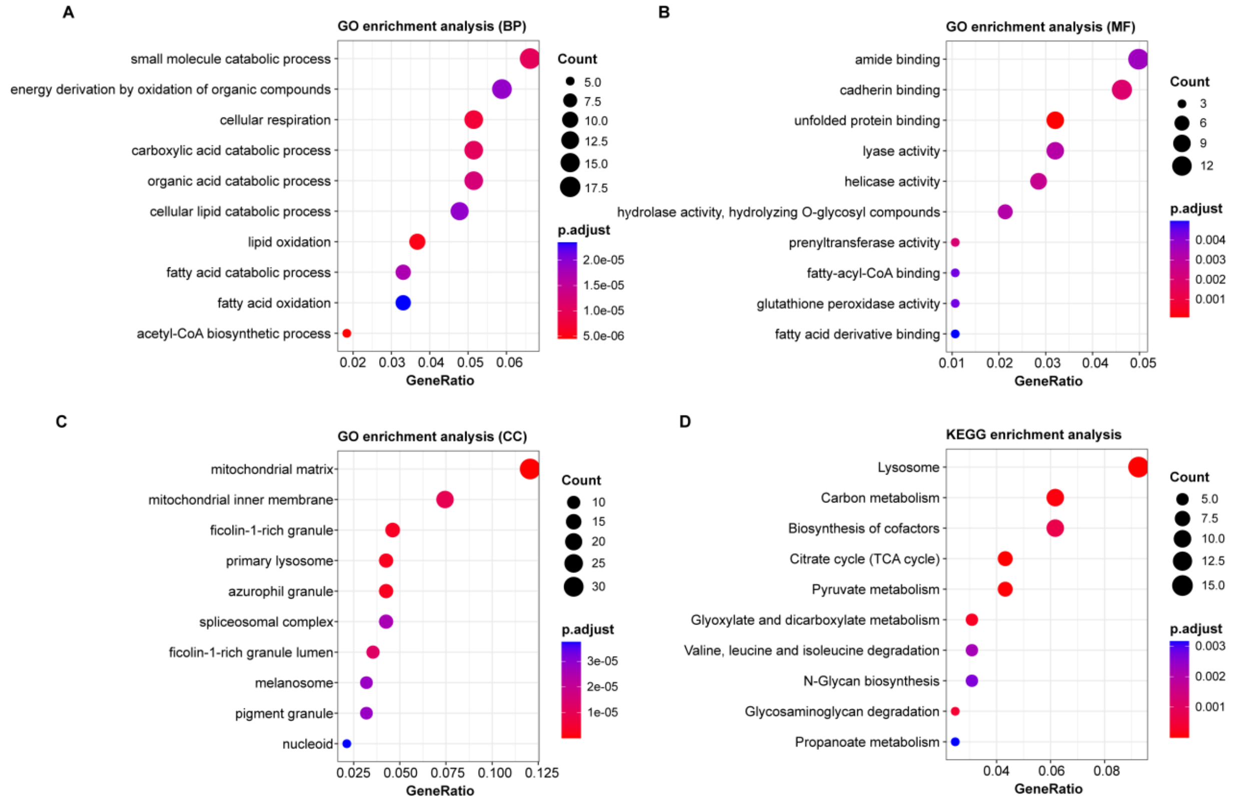

Figure S3 Correlation analysis of gene and clinical features. (A) Common clinical features. (B) Aecc_ali. (C) Batch_2. (D) Berlin_ards. * $p < 0.05$; ** $p < 0.01$; *** $p < 0.001$.

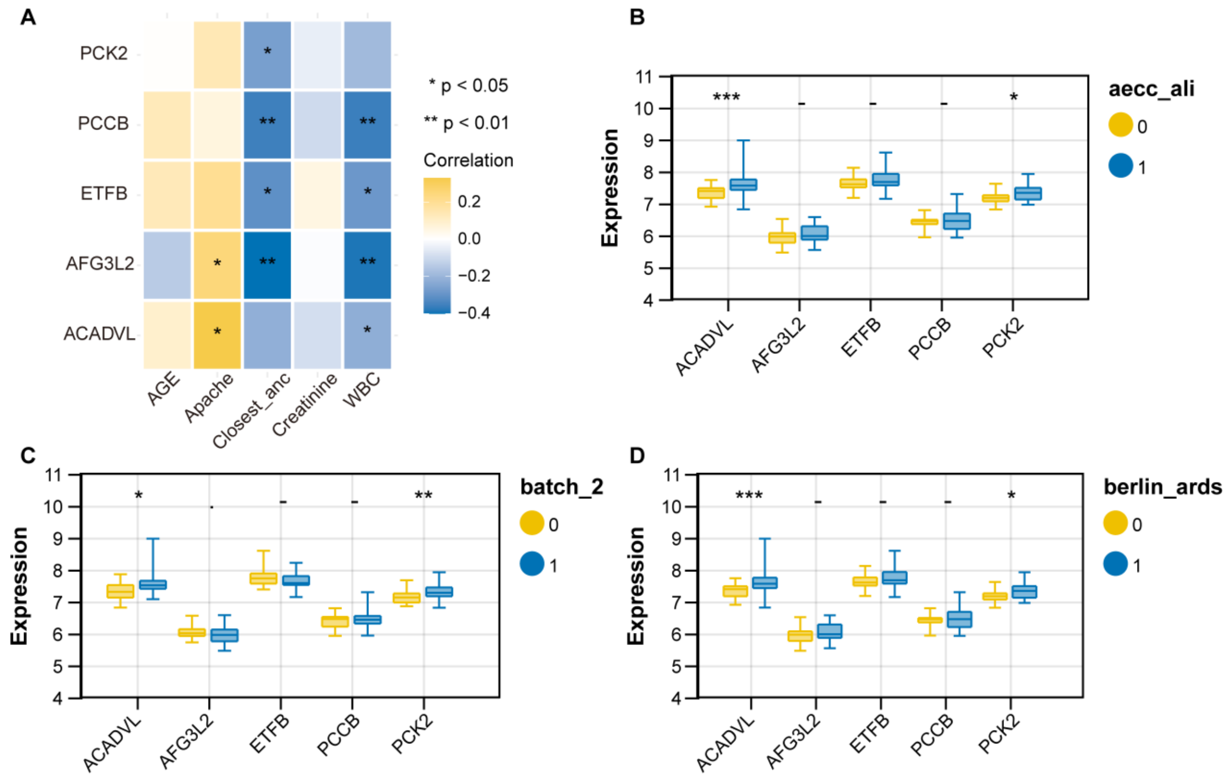

Supplement: Supplementary file 1 [file medi-104-e41497-s001.pdf]
